# The impact of rural-urban community settings on cognitive decline: results from a nationally-representative sample of seniors in China

**DOI:** 10.1186/s12877-018-1003-0

**Published:** 2018-12-29

**Authors:** Yuanxi Xiang, Hossein Zare, Cuiling Guan, Darrell Gaskin

**Affiliations:** 1 School of Management, Hubei University of Chinese Medicine, Wuhan, China; 2 Department of Health Policy and Management, Johns Hopkins Bloomberg School of Public Health, Hampton House Rm 441, 624 N. Broadway, Baltimore, Maryland 21205 USA; 3 University of Maryland- University College, Adelphi, USA

**Keywords:** Rural-urban, Community settings, Cognitive decline, MMSE, Multilevel modelling, China, CHARLS

## Abstract

**Background:**

Aging and rural-urban disparities are two major social problems in today’s ever-developing China. Much of the existing literature has supported a negative association between adverse community setting with the cognitive functioning of seniors, but very few studies have empirically investigated the impact of rural-urban community settings on cognitive decline in the late life course of the population in developing countries.

**Methods:**

Data of seniors aged 65 or above (*n* = 1709) within CHARLS (The China Health and Retirement Longitudinal Study, a sister study of HRS), a nationally representative longitudinal cohort (2011–2015) in China, were analyzed using a multilevel modeling (MLM) of time within individuals, and individual within communities. Cognitive impairment was assessed with an adapted Chinese version of Mini-Mental State Examination.

**Results:**

Urban community setting showed a significant protective effect (*β* = − 1.978, *p* < .000) on cognitive impairment in simple linear regression, and the MLM results showed it also had a significant lower cognitive impairment baseline (*β* = − 2.278, *p* < .000). However, the curvature rate of cognitive decline was faster in urban community setting indicated by a positive interaction between the quadratic time term and urban community setting on cognitive impairment (*β* = 0.320, *p* < .05). A full model adjusting other individual SES factors was built after model fitness comparison, and the education factor accounted for most of the within and between community setting variance.

**Conclusions:**

The findings suggest that urban community setting in one’s late-life course has a better initial cognitive status but a potentially faster decline rate in China, and this particular pattern of senior cognitive decline emphasize the importance of more specific preventive measures. Meanwhile, a more holistic perspective should be adopted while construct a risk factor model of community environment on cognitive function, and the influence at society level needs to be further explored in future research.

## Background

The looming demographic transition of China brings itself a major social and economic challenge, 10.8% of the population was aged 65 or above in 2016 [1]. It will only take 20 years (2017–2037) for China to double its percentage of the elderly population to 20%. This unmatched rate of aging is followed by 23 years in Japan, and 61 years in Germany historically [2]. There is also an institutionalized rural-urban division in China due to the establishment of a household registration system(hukou) in 1955. Industrialization and urbanization have drastically altered the rural-urban socioeconomic structure, China’s urban population exceeded its rural one for the first time ever in 2011. By 2016 the urban population was over 792 million accounting for 57.35% of the China’s population [1]. However, non-coordinated development still prevents the rural population from obtaining quality jobs, education, healthcare, housing, and other social determinants of health [3], and this disparity would impede China’s pursuit of becoming an industrialized country and global leader [4]. In the context of rapid aging and rural-urban disparity, more attention must be paid to the outcome of urban growth and its influence on human development and health status of the seniors.

Cognitive Stimulation Hypothesis suggests the lack of cognitive activities hastens impairment of cognitive functioning [5], and since senior and retired people usually spend a substantial amount of time in the community, they are more sensitive and dependent on the local resources and services where they live [6]. It is important to take potential determinants beyond individual-level factors into consideration in studying cognitive frailty, geographical variations in the prevalence of dementia indicate a possible effect of residential area settings on cognitive impairment in seniors [7]. While studying these environmental determinants and models of mental health problems, it’s critical to adopt a holistic and systematic perspective. A recent review had suggested a three-level conceptual framework of the pathway from community environment to the cognitive function of seniors [8]. Factors at individual, community and society levels might all contribute to cognitive frailty in later life. Poor quality of community environment could deprive seniors of basic activities, social interactions, and cognitive stimulation, and the cumulative stress it brought could undermine seniors’ cognitive functioning in the long term. Potential effects of meditation and moderation might exist at the individual level since different living environment might have various influences on people with different socio-demographic characteristics and health behavior and lifestyles. In a broader sense, societal factors, like politics, economics, geography, and culture might have substantial influence on community environments and cognitive function in a later life course as well.

There is an increasing trend of interest in exploring the association between community settings and the cognitive functioning of the senior population, a majority of the existing literature has been limited to a cross-sectional nature and suggested a possible negative association between them [9–13], by which causality between them cannot be implied. Almost no extant study adequately explores the cognitive decline of seniors in developing countries, and the unique socioeconomic structures of China, the pattern of social inequality might not as salient in China as previously tested in Western societies. The purpose of this study was to examine the impact of rural-urban community settings on cognitive decline in a nationally representative longitudinal cohort of seniors aged 65 or above in China. Since living environmental must be partially mediated through individual-level factors [8], the adjusted impacts of individual factors were also been assessed.

## Methods

### Study population

The China Health and Retirement Longitudinal Study (CHARLS) is a comprehensive study including assessments of social, economic, and health circumstances of community-residents, cognitive health is within in the module of health status and functioning. Participants were from the first three waves of CHARLS conducted in 2011, 2013, and 2015. The survey is part of a set of longitudinal aging studies that harmonized with the Health and Retirement Study (HRS) in the US so as to ensure best practice and international comparability. A multi-stage probability sampling method was utilized, 150 counties from 28 provinces were randomly selected with a probability-proportional-to-size (PPS) method from a sampling frame containing all counties in mainland China with exception of Tibet province. Administrative villages and community neighborhoods were used as primary sampling units (PSUs) in rural and urban areas respectively. Due to the complexity of the CHARLS survey which covers almost all aspects of personal life, a computer-assisted personal interview program (CAPI) was utilized to conduct the interview. The adoption of CAPI system can greatly help in time recording and item skipping while answering questionnaire and significantly reduce on-site errors. A final sample of 10,257 households including 17,587 participants aged 45 years or above was interviewed in the baseline [14]. In this study, participants who exited or deceased in follow-ups were excluded first, and only those participants who were 65 years old or above at the baseline and with complete data for the variables of interest were included. This reduced the sample to 1709 eligible observations.

### Measures

#### Community setting

The lowest level of administrative division in China is administrative village (cun) in rural or neighborhoods (shequ or juweihui) in urban [15]. The definition of rural or urban community type is based on the NBS’s (China’s National Bureau of Statistics) definition where a PSU is defined as urban if it is located in a city, suburb of a city, a town, suburb of a town, or other special areas where nonfarm employment constitutes at least 70% of the work force, and the rest is defined as rural [16, 17].

#### Dependent variable

Cognitive functioning was measured by an adapted Chinese version of the Mini-Mental Status Examination (MMSE) [18] with reference to the cognitive measurement conducted in the HRS [19], which tests 4 aspects of cognitive functioning with a full score of 32: orientation to time (today’s date, day of the week, and current season), recall (immediate and delayed recall of a list of 10 words), calculation (test of serial subtractions of 7 from 100), construct drawing (reproduce a picture of two overlapped pentagons). The score of every participant at each wave was graded by CAPI at site, and errors made in the examination (calculated as 32 - score) were used to indicate the cognitive impairment, a mean error number of 16.81 (standard deviation, 4.80; range, 3–30) was scored for the participants at baseline.

#### Independent variables

A recent published systematic review conducted to identify association between community environment and cognitive function had listed a series of potential effect modifiers at individual level [8], among which sociodemographic factors and health behavior such as age, sex, childhood residence type, subsidies from the government, occupation, education, and smoking habit were measured at the baseline survey in 2011. Even though this study has a specific enrollment age, there is emerging evidence that there should be an age-related gradient in performance of cognitive function, and cognitive decline should develop differently across sexes, and other intersectional characteristics under various circumstances [20, 21]. As there are accumulating evidence of impact of poor early-life conditions on older population’s health [22], and rural areas were so deprived of essential infrastructures and public services in the past, an item inquires where do you mainly live before 16 was used to indicate participant’s childhood residency type. The real individual and household incomes are difficult to acquire in China because of an in adequate tax system and the preference of using cash for payment. Hence the receipt of government subsidies (only targets low-income disadvantaged people, like disabled, aged, young, etc.) was used as an income indicator. Occupation was measured by two items in the questionnaire, first asks the type of your first job and second asks the ownership type of the business. Since China was in a centralized and planned economic system in the mid and late twentieth century, when job turnover was extremely low, and people employed within the state-owned or controlled business usually have higher salaries and better welfare. Therefore, occupation level was dichotomized into within or outside the state-owned business (“within” represents the better one). Educational attainment was measured as the highest level of education completed. Survey respondents could choose 11 options from illiterate to doctoral degree. For the convenience of this study, the answers were categorized into 3 levels. They are illiterate, elementary or equivalent, and middle school or above; Smoking is reported as an individual health behavior confounder of cognitive frailty [8], and its status was measured and categorized into never smoker, quit smoking, and still smoking.

### Analytic strategies

Simple linear regression analyses were utilized to estimate the bivariate association between independent variables and the dependent variable (Table 1). Multilevel modeling (MLM), also known as mixed-effect modeling, was used to explore the impact of rural-urban community settings on the cognitive decline of senior cohort over 3 waves to model time-variant change with unbalanced data (Table 2). The Three-level data structure includes data from baseline and 2 biennial follow-ups (level 1) clustered within 1709 individuals (level 2), clustered within rural-urban community settings (level 3). Previous studies showed the cognitive decline over time was non-linear, and a quadratic time effect was found [23, 24]. Level 1 modeled participants’ number of errors as a function of time, however, individual time terms tend to be correlated, and collinearity can undermine the stability of parameter estimates, the technique of orthogonal transformation was used to centered and scaled the two time terms of the second-order polynomial time function so as to make them independent [25]. Level 2 modeled individual-level socioeconomic and demographic factors, and level 3 modeled rural-urban community settings. This study used intercept (initial cognitive functioning) and first-order time slope (linear decline rate) as random effects at both level 2 and 3. The main effect of rural-urban community settings on cognitive decline was estimated after accounting for individual-level factors.

**Table 1:** Baseline Individual-level factors of the seniors and their bivariate association with errors in MMSE

Parameter	Number of Participants	%	β(SE)
Sex			
Male^a^	1043	61.03	
Female	666	38.97	1.455(0.235)***
Childhood Residency			
Village^a^	188	11.00	
City/Town	1521	89.00	3.031 (0.354) ***
Government Subsidies			
Yes^a^	882	51.61	
No	827	48.39	−1.417 (0.230)***
Occupation (State-owned Business)			
Outside^a^	1279	74.84	
Inside	430	25.16	−3.260 (0.256)***
Education			
Illiterate^a^	403	23.58	
Elementary school	856	50.09	−3.446 (0.258)***
Middle school or above	450	26.33	−6.230 (0.293)***
Smoking			
Never^a^	870	50.91	
Quit	240	14.04	−1.188 (0.349)***
Still	599	35.05	−0.270 (0.254)
Community Setting			
Rural^a^	1020	59.68	
Urban	689	40.32	−1.978 (0.232)***

*SE* Standard Error

^a^: This level was set as the reference level

** *p* < 0.01; *** *p* < 0.001

**Table 2:** Results from Multilevel modeling of community setting impact on cognitive decline of seniors in CHARLS

Mixed Effect	β(SE)						
	Model 1	Model 2	Model 3	Model 4	Model 5	Model 6	Model 7
Fixed Effects							
Within-Individual Covariate							
Intercept	18.493(0.132)***	−0.402(1.536)	− 0.403(1.536)	0.778(1.494)	9.511(1.456)***	9.282(1.447)***	8.691(1.456)***
Linear Time Slope	1.431(0.105)***	1.431(0.104)***	1.431(0.104)***	1.431(0.104)***	1.431(0.104)***	1.431(0.104)***	1.431(0.104)***
Quadratic Time Steepness	0.318(0.103)**	0.318(0.103)**	0.318(0.103)**	0.318(0.103)**	0.318(0.103)**	0.318(0.103)**	0.318(0.103)**
Individual-level Covariates							
Age (Year old) ^a^		0.229(0.021)***	0.239(0.021)***	0.232(0.021)***	0.148(0.019)***	0.160(0.019)***	0.163(0.019)***
Sex (Female)		1.845(0.195)***	1.885(0.195)***	1.638(0.190)***	0.553(0.184)**	0.575(0.182)**	0.163(0019)***
Childhood Residency (City/Town)		2.301(0.327)***	2.083(0.331)***	1.207(0.334)***	1.204(0.295)***	0.733(0.306)*	0.721(0.305)*
Government Subsidies (No)			−0.821(0.217)***			−0.470(0.194)*	−0.437(0.193)*
Occupation (Inside State-owned Business)				−2.579(0.247)***		−1.057(0.244)***	−1.069(0.243)***
Education ^b^							
(Elementary School)					−3.220(0.223)***	−3.121(0.222)***	−3.127(0.221)***
(Middle School)					−5.768(0.2645)***	−5.320(0.279)***	− 5.284(0.279)***
Smoking ^c^							
(Quit)							0.117(0.277)
(Still)							0.641(0.219)**
Community Setting(Urban)	−2.278(0.208)***	−2.100(0.212)***	−1.788(0.226)***	−1.381(0.216)***	−1.227(0.191)***	−0.830(0.207)***	−0.822(0.206)***
Urban × Linear Time Slope	−0.240(0.165)	− 0.240(0.165)	−0.240(0.165)	− 0.240(0.165)	−0.240(0.165)	− 0.240(0.165)	−0.240(0.165)
Urban × Quadratic Time Steepness	0.320(0.162)*	0.320(0.162)*	0.320(0.163)***	0.320(0.163)*	0.320(0.163)*	0.320(0.163)*	0.320(0.163)*
Random Variance							
Residual	10.841	10.866	10.867	10.872	10.872	10.872	10.872
Within Community							
Intercept/Initial Level	3.189	9.689	8.104	9.653	6.958	6.489	5.701
Time slope/Decline Rate	0.072	0.217	0.183	0.225	0.210	0.116	0.182
Between Community							
Intercept/Initial Level	11.025	2.299	3.750	1.384	1.566	1.837	2.551
Time slope/Decline Rate	0.248	0.052	0.085	0.033	0.048	0.141	0.075
AIC	29,533	29,320	29,307	29,216	28,908	28,884	28,878
BIC	29,618	29,424	29,418	29,327	29,025	29,015	29,022
χ^2^(df)		M2 vs M1 219.49(3) ***	M3 vs M2 14.27(1) ***	M4 vs M3 91.02 (0) ***	M5 vs M4 310.50(1) ***	M6 vs M5 27.97 (2) ***	M7 vs M6 9.62 (2)**

* *p* < 0.05; ** *p* < 0.01; *** *p* < 0.001

^a^age was measured as a baseline level at first wave

^b^illiterate was set as reference level

^c^Never smoker was set as reference level

To begin with, a base model was built that only contained orthogonal versions of one linear and one quadratic time terms, and total variance of cognitive impairment was partitioned into 3 sections: “between community setting”, “within community setting/between individuals”, and “within individuals”. Second, three individual-level factors, age at baseline, sex, and childhood residency type were added to the base model. Third, three individual-level socioeconomic factors, government subsidies, occupation, and education were successively added first and then all demographic and socioeconomic factors were simultaneously added up to the model. Finally, baseline smoking status was added up to make a fully adjusted model.

In terms of model fitting improvement comparison, AIC and BIC information indices were used. All analyses were performed in R version 3.4.3 using the “lme4” package (version 1.1–15), the orthogonal polynomial of second-order time terms were created by using R function “poly”, chi-square difference test was performed by using R function “anova”, and non-linear optimizer “bobyqa” was used to make model convergence more reliable [26].

## Results

Table 1 shows individual-level factors and rural-urban community settings of the study population at baseline. The age mean is 70.09 at enrollment, ranged from 65 to 90, with a standard deviation of 4.50. Male participants accounted for 61.03% of the cohort. Approximately 90% of the participants spent their childhood in city or town areas. Nearly half of the participants or their household received subsidies from the government in the past year, and only 25.16% of them worked at state-owned or controlled business. Over 3 quarters of them had an education level of elementary school or above. More than half of them self-reported as a never-smoker at baseline and almost 60% of them lives in rural community setting. Female sex, and city or town childhood residency were indicated as risk factors with significant positive slope estimates in the bivariate linear regression, whereas no government subsidies, occupation inside state-owned business, higher education level, quitting smoking habit, and urban community setting were indicated as protective ones with significant negative slope estimates. Details showed in Table 1.

Table 2 presents results of multilevel modeling of community setting impact on cognitive decline. In model 1, the first (*β* = 1.431, *p* < 0.001) and second (*β* = 0.318, *p* < 0.01) order time course terms are all significantly positive, indicating that time has a positive association with cognitive impairment. These estimates are robust with respect to model specification. Participants who resided in urban community setting had fewer baseline cognitive errors (*β* = − 2.278, *p* < 0.001) but faster quadratic rate of error growth (*β* = 0.320, *p* < 0.05) than did participants from rural community setting. In other words, senior residents in the urban neighborhood have better initial cognitive status but faster rate of decline. After controlling for individual-level factors of age, sex, and childhood residency type in model 2, urban community setting was still negative, and its quadratic rate of decline remained positive. Estimates of age (*β* = 0.229, *p* < 0.001), female sex (*β* = 1.845, *p* < 0.001), city/town childhood residency type (*β* = 2.301, *p* < 0.001) were still positive indicating worse initial cognitive statuses for these factors. Socio-economic factors of government subsidies, occupation, and education were added one after another in model 3, 4, and 5, estimates of these three factors remained negative which indicated that no government subsidies (*β* = − 0.821, *p* < 0.001), occupation inside state-owned business (*β* = − 2.579, *p* < 0.001) and higher education level (elementary school:*β* = − 3.220, p < 0.001; middle school: *β* = − 5.768, *p* < 0.001) were protective factors in accordance with the previous bivariate regression results. Compared to model 2, there was a 41.57% reduction in the estimate of urban community setting after holding education adjusted in model 5(model 2: *β* = − 2.100, *p* < 0.001; model 5: *β* = − 1.227, *p* < 0.001). Meanwhile, in model 7, a slight change 3.1% for elementary school level and 7.8% for middle school level in estimate reduction of education after adjusting for demographic and socio-economic factors.

Smoking habit was added to the model 7 as a health behavior factor, estimates of quit smoking and still smoking both changed into positive which is contrary to results of previous bivariate regressions, and the still smoking estimate is significant (*β* = 0.641, *p* < 0.01). After adjusting for all individual-level factors in model 7, urban community setting remained associated with fewer baseline cognitive impairment (*β* = − 0.822, *p* < 0.001) and faster quadratic rate of decline (*β* = 0.320, *p* < 0.05). The AIC and BIC indices decreased as more covariates were added up in subsequent models when compared with model 1(base model), and the relative improvement of the model fit was confirmed by chi-square difference test.

**Fig. 1:**
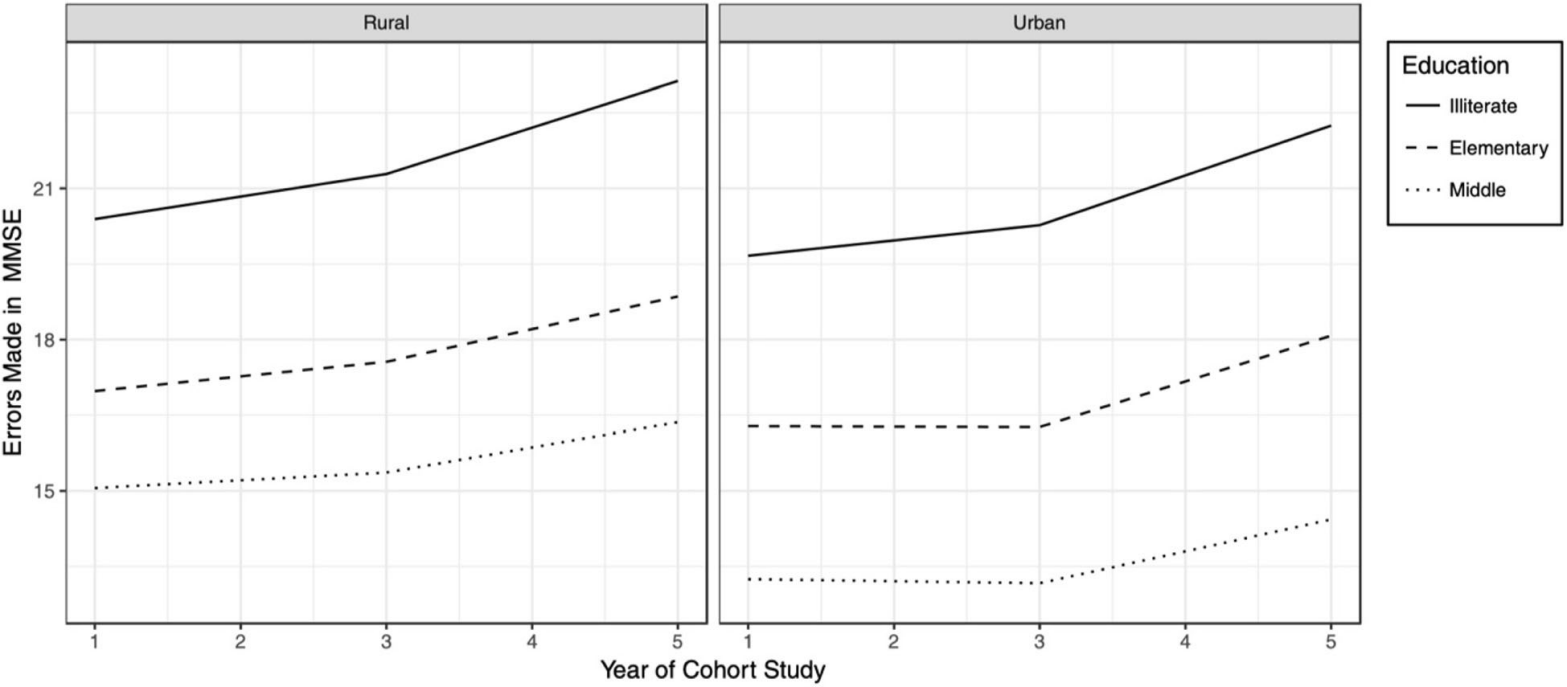
Growth Plot for Errors made in MMSE by seniors of different education levels from the rural or urban community setting over the three wave

Figure 1 plots the growth of cognitive impairment by seniors of different education levels from the rural or urban community setting over the three waves. In general, the rural participants had an overall higher errors number at baseline than the urban participants, which means their initial status of cognitive impairment was worse. However, the urban participants had a slightly steeper slope of error growth, in other words, the rate of their cognitive impairment was faster than the rural ones. Education Level had a considerable influence on the error growth, and the pattern was the same across two groups. The gap between illiterate and elementary school was wider than that of elementary and middle school, and the gap width seemed to remain some constant over the three waves.

## Discussion

Results from this study showed rural community setting was associated with poor cognitive initial status, but cognitive decline rate in urban community setting was faster than that in the rural one, and it did not seem to be changed after controlling individual-level factors. After adjusting individual-level factors of age, sex and childhood residency type, education level appeared to be the most prominent factor both in beta estimate and variance explanation. As it explained 28.2% of the within community variance and 31.9% of the between community variance for initial cognitive status when comparing model 2 with model 5. To the extent of our knowledge, this longitudinal study is the first to explore the impact of rural-urban community settings on the cognitive decline of nationwide seniors in China in last decade, therefore providing time insights on health and well-being of people in late life course under different environmental contexts in this population.

The underlying mechanism by which contextual condition may influence cognitive function is still not well established yet. Health is influenced by the complex interactions between environmental factors and body functions and structures as well as activities and participation. World Health Organization had developed a framework for healthy aging, called the International Classification of Functioning, Disability, and Health (ICF) [27]. As people aging, reductions will gradually happen in functional capacities, like walking, hearing, seeing, and cognitive ability. Furthermore, people’s contextual conditions change as they move in and out of different neighborhood over the life course. Even though in a limited time period, the characteristics of the neighborhood may change substantially including infrastructure and community development. To be specific, neighborhood may influence on personal mobility, sense of safety, potential chance of social interactions, access to healthy foods and green space, and exposure to pollution and crime [28]. Concerning poor physical functioning, seniors may have increasingly less time in motorized transportation and more time in the community [29]. Due to normal cognitive aging, seniors may be more vulnerable to community environment’s impact on physical ability and navigating difficulty [30]. In addition, the psychological health is also influenced by the social participation among seniors which provided by the community environment [31].

Urban community setting was consistently associated with better baseline cognitive function, even after adjustment of individual-level covariates. The similar evidence was also shown in other recent cross-sectional studies both domestic and international [12, 32–34]. Considering that China is a developing country with wide rural-urban disparities, urban areas have more supporting advantages of built environment, which provides easy access to food environment and local services [35]. However, the quadratic decline rate was faster in urban community setting than in rural one, this was inconsistent with some existing literature [24, 36, 37]. Since China is world’s most populous country and its drastic rise in urbanization in the recent decades, the downsides of high population density, high price in housing and accommodation, high cost of food and healthcare services, traffic congestion, etc. all lead to more and more constricted life space for the senior population, which has been shown to be associated with cognitive decline in literature [38, 39].

Having resided in urban settings as a child appears to be a risk factor for cognitive function, but urban setting has apparent protective effect on baseline cognitive functioning. This inconsistent effect of urban setting could be explained from sociohistorical aspects. Seniors aged 65 or over in 2011 were born before 1946, only 1 year after 2nd world war ended. During the war, most of the eastern and central China were invaded by Japanese army. Cities and towns where most governments and strategic resources located were fell. Contrary to the restless life in cities, rural areas were relatively safe. Illiteracy and low educational attainment have been shown to be a robust risk factor for cognitive impairment, and results in this study is consistent with other literature of developing countries [40]. The influence of smoking is inconsistent between the bivariate regression analysis and the multilevel modeling, this inconsistency is already reported, and the relation of smoking to cognitive impairment is possibly moderated or mediated by the presence of cardiovascular disease [41].

There are some limitations to acknowledge in this study. Although residential permit system and household registration system in China prevent people from migrating from place to place (mainly, from rural to urban) an individual could have lived in a deprived rural area for many years and moved to a privileged urban area temporarily, simply using the measurement of current community settings would not exclude such cases. Participants’ residential history should be more specified in future research. Second, the rural-urban community settings measurement may be only rough proxies of the built and social environment in community, to disentangle the effect of place from people, more specific contextual measurements, like social disorder, safety, ethnicity structure, public open space, food environment and local services, need to be included. Furthermore, more time point measures and follow-ups interviews data are needed to examine the long-term influence of community environment and interaction between place and people.

## Conclusions

This study provides empirical evidence that the neighborhood features of seniors might link with cognitive impairment in China. The findings showed a higher baseline level of cognitive functioning for seniors in urban areas but a faster decline rate than those in rural ones. The effect of place on health could be considered as a proxy for the quality of built and social environments in community. Due to the historical variation of economics, politics climate and culture in different societies, the built and social environments in rural or urban community settings were not consistently advantaged or disadvantaged. Since cognitive decline is a chronic process, the long-term effect of community settings on health needs more in-depth consideration at individual, community and society levels. Thus, the finding in this study reflected the China’s drastic changes of built and social environments in rural-urban communities in a life-long period, and more specific and different preventive measures should be implemented in these areas. Future research should be integrated with more advanced Geographical Information System (GIS) technology to assess the association between contextual conditions of neighborhood and mental health.

## Abbreviations

CAPI: Computer-assisted personal interview
CHARLS: The China Health and Retirement Longitudinal Study
GIS: Geographical Information System
HRS: The Health and Retirement Study
MLM: Multilevel modeling
MMSE: The Mini-Mental Status Examination
PSU: Primary sampling units
